# Anesthesia management for a child with the Koolen-de Vries syndrome: a case report

**DOI:** 10.1186/s12871-024-02508-7

**Published:** 2024-04-13

**Authors:** Yuyi Zhao, Yunxia Zuo

**Affiliations:** 1 Department of Anesthesiology, West China Hospital, Sichuan University, Chengdu, Sichuan, China; 2 The Research Units of West China (2018RU012)-Chinese Academy of Medical Sciences, West China Hospital, Sichuan University, Chengdu, China

**Keywords:** Koolen-de Vries syndrome, Rare disease, tracheo/laryngomalacia, Airway management

## Abstract

**Background:**

The Koolen-de Vries syndrome (KdVS) is a relatively new rare disease caused by micro-deletion of 17q21.31 which was first reported by Koolen in 2006. Typical phenotypes for KdVS include hypotonia, developmental delay, moderate intellectual disability, and characteristic facial dysmorphism. Up to now, there was only one case report about anesthesia management of patient diagnosed KdVS. It was a 2-year-old girl who experienced an MRI exam under anesthesia.

**Case presentation:**

We described a 21-month-old boy who planned to undergo an orchidopexy under general anesthesia diagnosed with KdVS. He had an intellectual disability, characteristic facial dysmorphism, tracheo/laryngomalacia, patent foramen ovale, and cryptorchidism related to KdVS. Due to the complex condition especially the presence of tracheo/laryngomalacia, we took some special measures, including reducing the amount of long-acting opioid, keeping the spontaneous breath, performing a caudal block, and applying the laryngeal mask. But the laryngeal mask was changed to an endotracheal tube because it failed to provide adequate ventilation. The boy experienced mild laryngeal spasm and hypoxia after extubation, but lateral position and etomidate eased his breathing problem and re-intubation was avoided. It is indicated that anesthesia management for patients with orphan disease is a real challenge for all anesthesia providers.

**Conclusions:**

The Koolen-de Vries syndrome is a relatively new orphan disease involving multiple systems. Keeping spontaneous breath, evaluating airway potency to anesthetics, applying endotracheal tube, and post-extubation lateral or prone position may be helpful for airway management for patient with hypotonia and tracheo/laryngomalacia. KdVS patient needs prolonged post-anesthesia monitoring and/or medication for airway complications.

## Background

The Koolen-de Vries syndrome (KdVS) is a relatively new rare disease caused by micro-deletion of 17q21.31 which was first reported by Koolen in 2006. Typical phenotypes for KdVS include hypotonia, developmental delay, moderate intellectual disability, and characteristic facial dysmorphism. Less common phenotypes include social and friendly behavior, epilepsy, musculoskeletal anomalies, congenital heart defects, urogenital malformations, and ectodermal anomalies [1]. KdVS patients may need sedation/anesthesia for diagnostic examination or surgery. Due to the low incidence and inexperience, KdVS creates anesthesia challenges. Up to now, there was only one case report about anesthesia management of patient diagnosed KdVS. It was a 2-year-old girl who experienced an MRI exam under anesthesia [2]. We here report a case that received surgery for orchidopexy under general anesthesia to provide experience in the management of this disease for other anesthesia colleagues.

## Case presentation

A 21-month-old boy presented to our center to receive an open orchidopexy surgery for a bilateral undescended testicle on March 18th, 2022. He was diagnosed with Koolen-de Vries syndrome when he was 6 months old. The child presented swallowing difficulty from birth, which showed that drank milk less and slowly. This symptom gradually eased after adding supplementary food at the age of 6 months old. He was diagnosed KdVS by gene test while the parents’ gene tests were negative. The involved gene KANSL1 is a protein-coding gene located at 17q. The mutation c.1183G> T(encoding p.Glu395*) revealed a heterozygous, pathogenic mutation. He was prone to have respiratory tract infections in winter and required oral medication or hospitalization. When he had bronchitis, he would have symptoms of stridor, which could be improved by putting a pillow under the shoulder. He had characteristic facial dysmorphism of KdVS, like tubular or pear-shaped nose, bulbous nasal tip, epicanthal folds, up-slanting palpebral fissures and telecanthus. He not only had the typical clinical features of KdVS including intellectual disability, and characteristic facial dysmorphism, but also tracheo/laryngomalacia, patent foramen ovale (PFO), and cryptorchidism related to KdVS. In addition, he also had hypothyroidism, indirect inguinal hernia, and syndactyly, which haven’t been reported associated with KdVS.

**Fig. 1:**
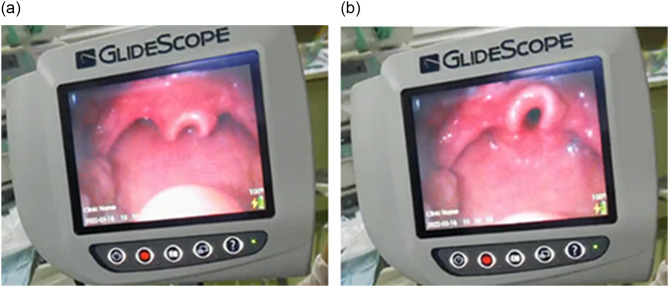
The epiglottis swung with spontaneous breath. (**a**) inspiratory phase; (**b**) expiratory phase

Prior to anesthesia induction, we re-checked his history, performed a physical examination, and monitored the ECG, SpO_2_, NIBP, and respiratory rate. He was 85 cm in height and 11 kg in weight. The vital signs were normal. He breathed calmly without symptoms of breathing difficulties, such as nasal flaring, or stridor. Due to the presence of tracheo/laryngomalacia, we planned to keep the spontaneous breath during induction to evaluate his tolerance to anesthetics. Atropine 0.25 mg was administered intravenously for reduction of the secretion. Then we administered midazolam 0.75 mg, fentanyl 20 µg, and etomidate 6 mg as well as dexamethasone 2 mg infused in 2 min intravenously. Then, the patient inhaled sevoflurane from 1 to 3%. The tidal volume of spontaneous breath was adequate initially, but gradually decreased with the deepening of anesthesia. The patient showed breathing difficulty with the suprasternal fossa retraction significantly in the inspiration period and bulging at expiration. Immediately we started manual ventilation by CPAP maintaining Peep at 5cmH_2_O. The obstruction was soon solved and the tidal volume improved. After inhaling 3% sevoflurane for about 10 min, we tried to establish an artificial airway. We inserted a glidescope which showed that the epiglottis was long, curved, and swinging with the breath (Fig. 1). Then we inserted a gel-laryngeal mask guided by a glidescope to make sure it was in the right position. But the inspiratory pressure was high up to 22 cmH_2_O with the tidal volume only about 30 ml during mechanical ventilation. So we changed the laryngeal mask to a 4.0^#^ endotracheal tube. Then the ventilation parameters improved significantly with ideal airway pressure and tidal volume. Anesthesia was maintained by inhaling 3% sevoflurane and intravenous infusion of remifentanil 0.1-0.15ug/kg/min. A caudal block with 12 ml 0.5% ropivacaine was performed for intraoperative and postoperative analgesia. The open orchidopexy operation lasted for about 80 min. Since muscle relaxant was not used, spontaneous breath quickly returned after sevoflurane inhalation and remifentanil infusion terminated. When the child showed signs of body movements indicating he was awake, we removed the endotracheal tube. The child was very irritable and seemed difficult to breathe with obvious suprasternal fossa retraction, and the color of his mouth turned blue rapidly. We began mask ventilation immediately, but the situation didn’t improve, and SpO_2_ went down to 84%. So we turned the child to the right lateral position and intravenously injected 2 mg etomidate. The patient gradually calmed down, the color of his mouth became red, and SpO_2_ went up to 100% within 90 s. But stridor still existed. After 10 min monitored observations, the child was transported to post anesthesia care unit (PACU). The child preferred to lie prone in the PACU. During 48 h after the anesthesia, the suprasternal fossa retraction and stridor existed continuously and optimized on the 3rd day after the operation. The parents reported that the child recovered well in the follow-up a month later.

## Discussion and conclusions

Open orchidopexy surgery can be performed under spinal anesthesia or caudal block. Either of them has to be combined with general anesthesia because this child is 21-month-old. We are more familiar with caudal block which is almost conducted in all open orchidopexy surgeries in our hospital. Therefore we performed caudal block instead of spinal anesthesia. Due to the special facial appearance, hypotonia and tracheo/laryngomalacia caused by KdVS, children may have difficulty in ventilation and intubation. To prevent airway emergencies and preserve spontaneous breathing, we took some precautions. Firstly, we reduced the amount of long-acting opioid and slowed down the induction duration. The caudal block was performed to compensate for the insufficient analgesia caused by the reduction of long-acting opioid. Secondly, all the induction anesthetics were carefully chosen and administered cautiously. We chose etomidate instead of propofol because previous studies had shown less effect on respiration of etomidate compared to propofol [3]. Sevoflurane was selected and initiated from a low concentration of 1% increased by 0.5% every 10 to 20 breaths up to 3%. All the above measures were designed to test the effect of anesthetic drugs on the child’s airway potency after sedation. In the previous report about a KdVS patient who received an MRI exam under sedation, Ali mentioned the potential risk of increased sensitivity to nondepolarising neuromuscular blocking agents and the depressant effects of anesthetic agents related to hypotonia [2]. It had been proved that the child’s airway potency did progressively deteriorate as the sedation level elevated. If we didn’t keep the spontaneous breathing during anesthesia induction, a high risk of malignant hypoxic events might be developed by difficult ventilation. Extubation can be performed under deep sedation or awake. Since this child had tracheo/laryngomalacia and experienced difficult breathing during anesthesia induction, we thought it was beneficial to extubate awake. That was why we chose awake extubation. We removed the endotracheal tube when the child had body movements. Even if the above preparations and plans were made, oxygen desaturation still occurred when we removed the endotracheal tube during anesthesia recovery. This might be caused by the combined effects of tracheo/laryngomalacia, hypotonia related to KdVS as well as partial laryngeal spasm. Perhaps the child’s movement was not purposeful which meant he was not awake when we extubate him. We might have extubated him during light anesthesia. Etomidate instead of propofol or muscle relaxants was chosen to treat laryngeal spasm which did improve the airway obstruction and oxygenation in some extent. Lateral position further eased his breathing and re-intubation was avoided. He preferred prone position in PACU indicating deep extubation can be problematic. Perhaps extubation in deep anesthesia with lateral position may somehow prevent hypoxia.

Because of the less stimulation of the laryngeal mask which allowed reduction of anesthetics and free of muscle relaxant, and advantages for difficult airway management compared to endotracheal tube, we took it as the first choice for airway management. Previous studies had shown that inflatable and non-inflatable laryngeal masks could provide equal ventilation quality, and the i-gel presented a better sealing effect [4, 5]. Meanwhile, the gel-laryngeal mask is the most commonly used LMA in our center which usually provides very high seal pressure as good as inflatable LMAs. So we chose gel-laryngeal mask for this case. But it failed to provide adequate ventilation. The result confirmed that the endotracheal tube can also be inserted without muscle relaxant and provide better ventilation support. This suggested that an endotracheal tube may be more appropriate compared to a laryngeal mask for patients with hypotonia and tracheo/laryngomalacia. After extubated awake, he still had stridor and inhaled acetylcysteine (3 ml: 0.3 g) for 3 days to release the airway symptoms. This reminded us that prolonged postoperative monitoring and prevention of respiratory complications were necessary for these patients.

In addition, this patient also had hypothyroidism, indirect inguinal hernia, and syndactyly. Since KdVS is a relatively new discovered rare disease, it is unclear whether these co-existed diseases are related to KdVS. For our patient, these problems were controlled in the stable status which won’t significantly increase the perioperative risk. KdVS involves multiple systems and variable clinical features, such as growth retardation, seizures, structural central nerve system anomalies, neuropsychological disorders, scoliosis/kyphosis, hearing impairment, congenital heart anomalies, ectodermal abnormalities [1]. It is necessary to perform a comprehensive preoperative exam for these patients.

The specific facial dysmorphism, tracheo/laryngomalacia and hypotonia associated to KdVS makes airway management a challenge for anesthesia providers. Keep the spontaneous breath during induction period and carefully evaluate the effect of sedation to airway potency may be the key techniques. Endotracheal intubation is perhaps more suitable than laryngeal mask for such children, which can also be conduct with low-dose opioids and without muscle relaxants. Regional anesthesia can reduce the use of opioids. We should extubate awake, then a lateral or prone position may improve ventilation. The Koolen-de Vries syndrome involves multiple systems, thus individualized anesthesia protocol should be designed following a comprehensive evaluation.

## Data Availability

Data sharing is not applicable to this article as no datasets were generated or analyzed during the current study.

## Abbreviations

KdVS: Koolen-de Vries syndrome
PFO: Patent foramen ovale
CPAP: Continuous positive airway pressure
PACU: Post anesthesia care unit
